# Truths and Facts

**Published:** 1876-04

**Authors:** 


*Truths and Facts.*—In the very able address of the Rev. A. C. Evans, published last month in the RECORD, our attention was particularly called to the distinction he demonstrated between "truths and facts." It was the more striking as we find collateral illustrations of this difference every day in the lives of men, and their various pursuits and relations. As it is our wish that the thoughts here suggested may be productive of some good to our readers, we hope to be pardoned for devoting so much space to these reflections. While facts are frequently momentous things, truth, even sometimes independent of facts, is of more importance, and it is a lamentable truth that mankind is more influenced by facts than truths. It is to be deplored that men look more frequently at the actions of others than the motive that prompted the acts. The motive is the truth hidden beneath the action—the main spring that irresistably forced it into operation before the gaze of men; and to detect and reveal this hidden spring is of more MORAL importance than the other. We are prone to be influenced by an act rather than the motive, because an action is a thing that we can *see*, but it often requires earnest search, and a delicate, sympathetic and impartial discernment to discover the truth or motive power from which the act was evolved. To do this is God-like, for as some writer has said: "Men judge us by our actions; God looks at the motive." If this rule (the one God-like) was individually and universally carried out in our moral codes, justice would more frequently take the place of its antipodes; truth would oftener prevail where error or guilt, under the mask of facts, triumphs and laughs in the face of the world; and the bitter feeling engendered in some struggling, tempted soul by misconstruction of an action would be changed to noble pride and unalloyed gratification.

But we see it demonstrated every day that men with the most ignoble and corrupt motives, can gain honors, applause and high places; while the truly noble man, in whose life Truth shines out like a star in a cloudless sky—whose integrity is as unshaken as the granite mountain base—is neglected, scorned and unappreciated—is unsought and unhonored by the world. In both these instances we see the facts, and are governed by them. In the former, the man has perhaps acquired wealth, and, thereby, position; he is solicited to accept government offices or public enterprises, and may, also, be "a pillar" in one of our Christian churches. These things are all facts; we see them plainly enough; but *how* he gained his wealth and position—these honors and emoluments—this fawning homage from society—men and the church can only be answered by the *truth* underlying these *facts*. This is one of the many cases where we pretend not to "discern the truth." We know that this man is an unmitigated *fraud;* we know that his motives are corrupt, and that he is a hypocrite of more than Judas assurance; we know he is a thief, branded so by his conscience and the revelations that have been made against him; and we know that he is not capable of honorably and honestly filling the places to which he aspires and is appointed, and that, if justice were meted out, he would be the inmate of a felon's cell. But, because of the "facts" mentioned above—frequently a mere quibble or technicality—we trample upon truth that is struggling to come to light, and prostitute our conscience and self-respect to the honorable (?) privilege of bending the sycophant's knee to a colossal image moulded of "the precious ore." Yet, we often hear some of these very persons, who do homage to such "golden calves," deploring the decrease of vital religion in the church, the speculations of various officials, and the demoralizing tendencies among us.

This corrupting feature of the moral world is obvious among all pursuits and professions. Alas, even some ministers of the Gospel have their "golden calves," and from the pulpit will denounce the facts that make a man's life unrighteous; and then, again, for fear of being unpopular with their wealthy brethren, will put our holy religion to open shame, by skillfully ignoring these facts, either from the sacred desk, or by some of the words and acts of their daily lives; thus failing to lead their people to be impartial discerners of the truth, and to follow in the footsteps of Him who is no respecter of persons.

The fact that a man has won pecuniary success; that he has done some good deeds, and for a short time has had a brilliant career, does not prove that, at heart, he is sound, and that his motives were God-like. For a year or two he may have the reputation of being an honest man, and if by slipping aside from the right, he loses that character, with the dextrous trickery of the juggler, he changes his position and becomes prominent in charitable movements, public enterprizes, etc. But at bottom, he is a moral coward; he has "flinched when most of all should have been true," and it is but a question of time when his great mushroom reputation will be utterly demolished. A temporary reputation may be gotten up upon manufactured facts—but a good character must emenate and live upon truths. A willful slanderer can no more be a gentleman than a thief can be a Christian, though the facts presented may not in strict accordance with the law prove him guilty. The endorsers of such characters, with a knowledge of truths that would prove them guilty and imprison them in the penitentiary, are to be pited, and never should be trusted, not even with facts, as they will always use them to either crush the truth or vindicate it as it may appear to their minds most likely to advance their interest. It is a sad truth that the number of bad men who rob the country of money and honor; who destroy the power and influence of our church organizations, contaminate society, and demoralize our children, and who manage to escape punishment on an array of technical facts, are rapidly on the increase, because of the depreciation of the truth—the low public estimate that is now placed upon character, and the great advance in appreciation and importance of facts—facts that legally prove the truth to be false, and protects the guilty criminal from prosecution. It is regretted by all good men that a few of those creatures who disregard the truth and who rely on facts to carry them safely and triumphaetly through this life, have crept into our Heaven-born profession; hence the importance of disseminating the important truth contained in the very beautiful, able and unusually original address of our learned Christian friend, General C. A. Evans. I trust that it will be read and reread by our readers.

In the valedictory address of Dr. Adams, also published in the last number of THE RECORD, we were again led to some reflections, *apropos* to the subject, by a sentence in which the talented young physician says: "Character is life, and he who would make his life great, must make his character noble." Grandeur of character does not consist in doing great and heroic deeds, as the world goes. A noble character is elevated more from the inner than the outer life, and generally the noblest, most heroic deeds are done in such obscurity and privacy, they are unrecorded by the world, and are frequently known only to the doer and the all-seeing eye of God. There the world fails to discern the grand truth that he is the greatest hero who, with a manly spirit and dauntless courage, meets the responsibilities of his lot; who struggles against and triumphs over temptation, alone and unaided by any human sympathy; who holds fast to his truth and integrity despite obloquy, shame, poverty, and even bitterest want; and who, unyielding and immovable in the faith of right, "will dare to be unpopular, to be neglected and misapprehended—to follow the voice of God, though it leads into the wilderness, and to tell the Devil to his very face that he lies."

Carlyle has said that it requires more courage to live than to die. But "how many men lack this courage! They are ready to die; the dark passage has no terrors for them; their faith will carry them triumphantly through. Yet, when it comes to the question of living, they fail. They dare not be true to themselves; they fall in with the tide of evil that sweeps around them. A gigantic wrong exists, but all that is dearest to them is inextricably intertwined with it, and to try to overthrow it would sunder their heart-strings. Why not, then, let it go on? To do the right thing they believe will entail upon them poverty, neglect and scorn. What is the use of doing it when, by swerving just a little from the right, they can have love, obedience, wealth, and troops of friends? How many reason thus, and fall! They dare march to the cannon's mouth, or meet loathsome disease, but they dare not do what they are thoroughly convinced is right."

We want men in all professions and conditions who have the courage to live; who will hold fast to the truth, "though all is dark, except where God shines in; who will be faithful though the heavens fall, and hearts are broken and friends forsake." We want a host of such men—a grand innumerable army that will present a solid front to meet the wrong and evil that is fast devastating the pure beauty of moral and religious dominions, Oh, that the truth might prevail in our social and home circles, in our finances and commerce, in all labor and professional careers, in church and state; God grant us the courage to live to tell harsh truths; to overthrow splendid falsehoods; to disown sweet lies, and to fling aside honors, wealth and the praise of friends, rather than impair for a moment the soul's integrity."

From the many indications in the moral world, it appears to be the turning point in its existence, the fiat that shall decide whether its resplendent glory, now veiled in gloom, shall again illuminate the path of mankind, or it shall be thrown out of its orbit into dark and trackless chaos. God seems to be weighing this sphere of men's souls in the balance, and the slightest tip of the beam may hurl it to irreparable destruction. To avert this doom, men must now be true themselves, if they never have before;. they must come forward *en masse*, with a force superior to that of all demoralizing agencies, and throwing themselves into the opposite scale, decide the momentous question. Men never had more splendid opportunities of becoming heroes in the moral world than now, and "the man lives twice who lives the first life well."

To the readers of THE RECORD we would especally appeal in behalf of this moral crusade. The "ministry" of the Healing Art is only second to that of the Gospel; and of all men, next to the embassadors of the most High, the physican should demonstrate the elevation of human character. If the chairs in all our medical colleges were filled with men of high moral culture and learning, what an exaltation it would give the profession, and with what pride and confidence men would place their sons under the guidance and instruction of those who were truly architypes for the medical novitiate. But, as is sometimes the case, the friendship and services of bad men in the profession will be preferred to those of an honorable gentleman and true physician. Some friends of wealth and position may, therefore, forsake you, but let them go; remember that if your life is pure and noble, these *facts* are continual and sharp reproofs to some men, consequently they will shun you as a companion, and ignore your professional skill. The loss of such "profitable patrons" will be supplied by others who are capable of appreciating your personal worth and your "service to suffering man." All *pure and worthy* men and women will honor you as exponents of a noble manhood, and place upon your brow the crown that is due the physician who exalts human character, and that profession which, in itself, is a noble TRUTH. P.

